# Parasitic Reflection Eliminating for Planar Elements Based on Multi-Frequency Phase-Shifting in Phase Measuring Deflectometry

**DOI:** 10.3390/s24041239

**Published:** 2024-02-15

**Authors:** Siya Huang, Yuankun Liu, Xin Yu

**Affiliations:** College of Electronics & Information Engineering, Sichuan University, Chengdu 610065, China; huangsiya@stu.scu.edu.cn (S.H.); xin_yu@stu.scu.edu.cn (X.Y.)

**Keywords:** phase measuring deflectometry, parasitic reflection, multi-frequency method

## Abstract

Phase measuring deflectometry (PMD) stands as an extremely important technique for specular surface measurement. However, the parasitic reflection from the rear surface poses a challenge for PMD. To solve this problem, this paper proposes an effective method based on multi-frequency and phase-shifting to search for the correct phase. Firstly, the relationship between the phase error and fringe frequency is adequately investigated. Subsequently, an auxiliary function is established to find the special frequency at which the phase error is zero theoretically and the unwrapped phase is the phase of the top surface exactly. Then, the shape of the top surface can be reconstructed correctly. A standard plane element with a thickness of 40 mm and a flat glass with 19 mm were measured. The experimental results verify the feasibility of the proposed method. Considering the result of the interferometer as a reference, the RMSE of the error map is up to 20 nm for the standard plane element. The experimental results demonstrate that the proposed method can successfully untangle the superposed reflections and reliably reconstruct the top surface of the object under test.

## 1. Introduction

Among optical three-dimensional measurement techniques, phase measuring deflectometry (PMD) is an efficient, flexible, and rapid full-field measurement technique based on the law of reflection. This technique is widely applied for the surface measurement of mirror or mirror-like objects, whose accuracy is nearly equivalent to that of interferometry [1,2,3,4]. And PMD works well for single-side reflection objects of different sizes, in monocular, binocular, or even more cameras, with one screen or two screens, and so on [5,6,7,8].

However, when dealing with transparent objects, the reflections from the top and bottom surfaces are captured by a camera simultaneously. In this condition, the deformed patterns are the superposition of the fringes, resulting in the failure of reconstruction. Usually, the reflection from the bottom surface is called parasitic reflections [9]. To solve this problem, some strategies are proposed which make modifications or alterations at the hardware level. One typical method is to roughen or blacken the bottom surface, but it is usually not preferred due to its destruction. Another solution is to employ ultraviolet light as an illumination source. But this method entails considerable costs and challenging technical difficulties [10].

Other strategies primarily involve improvements within the software algorithms. In principle, the reflections from the top and bottom surfaces can be separated spatially. A class of methods is to directly avoid forming the superposed fringes, in which a binary pattern or gray code is used to separate the overlapping signals [11,12]. The former needs to set a suitable size of Zone-M to cover the measurement area [11]. The latter needs to know the phase-shift caused by the thickness difference between the upper and lower surfaces [12]. Another is to improve the algorithm to extract the phase from the superposed patterns. Faber et al. proposed a multi-frequency method that used the difference in the data at different fringe frequencies to solve a fixed set of unknowns to decouple the superposed fringes [13]. Based on the multi-frequency approach and combined with Fourier-transform, Wang et al. converted the intensity sequence into the Fourier domain to analyze it. Then they obtained a conclusion that the two isolated peaks in the Fourier domain corresponded to the screen coordinates of the top and bottom surfaces and thus utilized it to reconstruct the top surface. Its accuracy is subject to the resolution in the Fourier domain [14]. In practice, the existence of optical aberrations causes the attenuation effect that the modulation intensity reduces with the increase in fringe spatial frequencies. In order to get closer to reality, scholars take this effect into consideration subsequently. Tao et al. proposed an envelope curve algorithm based on the multi-frequency method. This method modeled the attenuation effect with an exponential modulation transfer function (MTF) [15]. Considering the attenuation effect as a Gaussian low-pass model, Ye et al. solved the unknowns in groups, alternately optimizing them. Finally, both surfaces of a lens were reconstructed simultaneously using a hybrid reflection–refraction model [16]. And Zheng et al. adopted the same Gaussian low-pass model to add the intensity model. They calculated the initial coordinates of the front and back surfaces through a power spectrum and optimized the model with the aim of minimizing the difference between the measured intensity values and the model values. Then the front and back surfaces could be obtained [17]. However, the two algorithms are required to use the inverse ray-tracing method [16,17]. Combining the multi-frequency method and phase-shifting technique, Leung et al. proposed an algorithm without modeling the attenuation effect. But only a 6 mm × 6 mm region was measured [18].

In order to extract the phase precisely from the superposed patterns, to the best of our knowledge, this paper, which mathematically models the relationship between the fringe frequencies and the phase errors when parasitic reflection exists, proposes a new multi-frequency phase-shifting approach to find an optimal phase value rather than searching for the difference between the two light source pixels on the screen. By analyzing the characteristics of the phase error model and introducing an auxiliary function, the special fringe frequencies with zero phase errors can be found. After the true phase of the top surface of the transparent object is extracted, an accurate measurement will be fulfilled. The remainder of this paper is organized as follows: Section 2 introduces the principle of the PMD and the proposed method. Section 3 presents the experimental results of the proposed method. A discussion of this method is hosted in Section 4. Section 5 concludes the work for this whole paper.

## 2. Principle

### 2.1. Phase Measuring Deflectometry Principle

**Figure 1:**
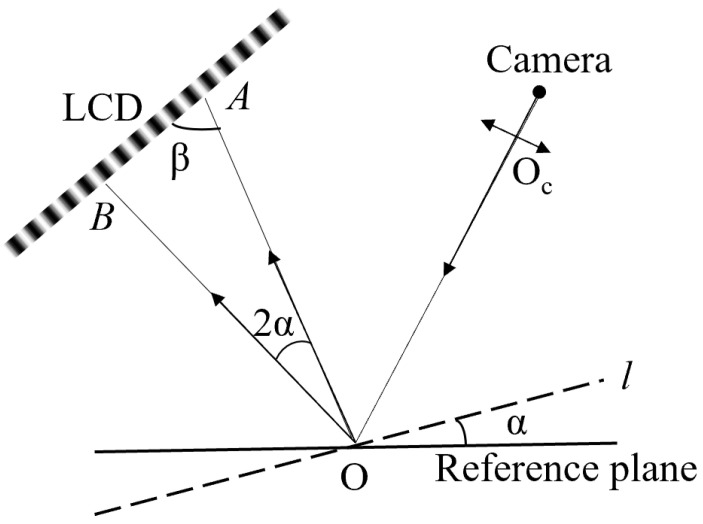
The measurement principle of PMD.

Here, we explain the fundamental principle of PMD from the point of view of ray reflection. As shown in Figure 1, an angle change in the surface under test, i.e., plane *l*, with respect to the reference orientation, is *α*, which causes the reflected ray to rotate by angle 2α. Apparently, a pixel on the camera image plane will see the two points, i.e., *A* and *B*, of the display screen via the reference plane and plane *l*, respectively. The distance $\bar{AB}$ between *A* and *B* is related with the change in the incident angle, which can be expressed as:

(1) $$\frac{\bar{AB}}{\sin2\alpha }=\frac{\bar{OA}}{\sin(\pi -2\alpha -\beta )}$$

Furthermore, the phase values of points *A* and *B* can be denoted as $\Phi_{A}$ and $\Phi_{B}$, respectively. Then, $\bar{AB}$ can be also expressed as follows:

(2) $$\bar{AB}=\frac{\left|\Phi_{A}-\Phi_{B}\right|}{2\pi f}=\frac{\Phi_{AB}}{2\pi f}$$

where *f* is the fringe frequency and $\Phi_{AB}$ is the difference value between $\Phi_{A}$ and $\Phi_{B}$. Combined Equations (1) and (2) are given by

(3) $$\tan2\alpha =\frac{\Phi_{AB}\sin\beta }{2\pi f\cdot \bar{OA}-\Phi_{AB}\cos\beta }$$

When $\bar{OA}\gg \bar{AB}$ and the angle α is quite small, there are $\bar{OA}\approx \bar{OB}=L$ and β≈90°, approximately. Then, Equation (3) can be rewritten as

(4) $$\tan\alpha =\frac{\Phi_{AB}}{4\pi fL}$$

This equation shows the relationship between the phase values and the surface slopes. The orthogonal slopes can be obtained by decoding the deformation fringes in horizontal and vertical directions, respectively. Then, the tested surface can be obtained by integrating the surface slopes using the wavefront reconstruction algorithm [19,20,21].

However, the phase of the top surface cannot be directly calculated in the presence of parasitic reflection from the bottom surface of the transparent object. Therefore, a new method is proposed in this paper to solve this problem.

### 2.2. Phase Extraction with Parasitic Reflection

#### 2.2.1. The Phase Error Model

**Figure 2:**
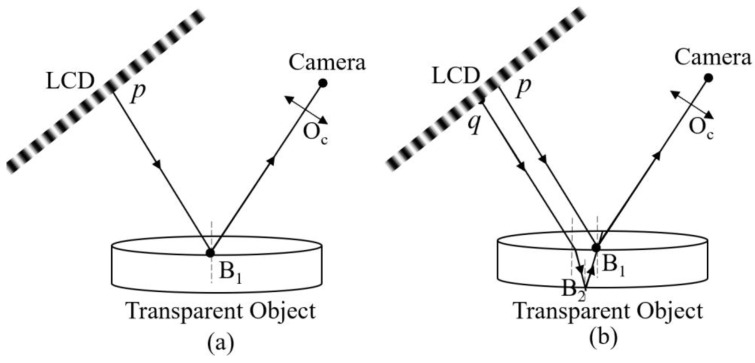
The system of PMD (**a**) without parasitic reflection; (**b**) with parasitic reflection.

In PMD, as shown in Figure 2a, the light intensity captured by a camera pixel can be expressed as follows:

(5) I(u,v)=A(u,v)+M(u,v)cosϕ(u,v)

where $A\left(u,v\right)$ is the background intensity, $M\left(u,v\right)$ is the fringe modulation intensity, and ϕ(u,v) is the phase distribution of the fringe. For convenience, the notation (u,v) will be omitted from the equations detailed hereafter.

With parasitic reflection, the light intensity captured by a camera pixel can be rewritten as follows:

(6) $$I=A+M_{1}\cos\phi_{t}+M_{2}\cos\phi_{b}$$

where $M_{1}$ and $M_{2}$ are the modulation intensities related to the top and bottom surfaces, respectively; $\phi_{t}$ and $\phi_{b}$ are the fringe phases related to the two surfaces, respectively. If the phase-shifting amount $\delta_{n}=\frac{2\pi n}{N}$ (where n=0, 1,…, N−1 and N≥3) is introduced, the light intensity measured by a camera pixel at the nth phase-shift can be expressed as:

(7) $$I_{n}=A+M_{1}\cos(\phi_{t}+\delta_{n})+M_{2}\cos(\phi_{b}+\delta_{n})$$

This is according to the orthogonality of trigonometric functions, given by

(8) $$\left\{ \begin{array}{l} M_{1}\sin\phi_{t}+M_{2}\sin\phi_{b}=-\frac{2}{N}\sum_{n=0}^{N-1}I_{n}\sin\delta_{n}\\ M_{1}\cos\phi_{t}+M_{2}\cos\phi_{b}=\frac{2}{N}\sum_{n=0}^{N-1}I_{n}\cos\delta_{n} \end{array}\right.$$

According to Reference [18], when the angle of incidence $\theta_{in}$ satisfies $0^{{}^{\circ}}\leq \theta_{in}\leq 60^{{}^{\circ}}$, the relationship of $M_{1}$ and $M_{2}$ can be expressed as

(9) $$M_{2}=\gamma M_{1}$$

where *γ* is a ratio and 0<γ<1. There is no parasitic reflection anymore when *γ* = 0. In this situation, the measured phase is the true phase $\varphi_{w}$ of the top surface, and it can be given as

(10) $$\varphi_{w}=-\arctan\frac{\sum_{n=0}^{N-1}I_{n}\sin\delta_{n}}{\sum_{n=0}^{N-1}I_{n}\cos\delta_{n}}$$

When γ≠0, the measured phase is the comprehensive result originated from the reflection of the top and bottom surfaces, given by

(11) $$\varphi =\varphi_{w}+\Delta \varphi$$

where ∆φ is phase error caused by parasitic reflection. As shown in Figure 2b, a camera pixel will collect the two light rays, which are reflected from the top and bottom surfaces of the transparent object. The corresponding points on the screen are *p* and *q*, respectively. Generally, the distance between *p* and *q* needs to be decomposed along the fringe directions. Without the loss of generality, we take the *x* direction as an example.

**Figure 3:**
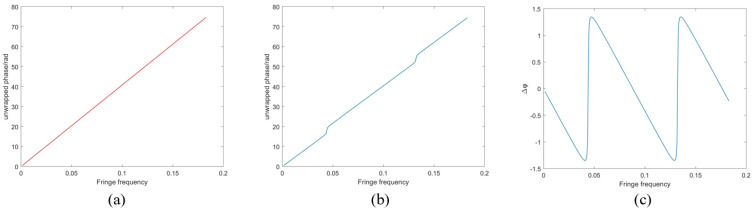
(**a**) The continuous phase without parasitic reflection; (**b**) the continuous phase with parasitic reflection; (**c**) the variation in phase errors with frequencies.

Due to the linear relationship between the continuous phase and the screen coordinate, as shown in Figure 3a, the continuous phase can be expressed as ϕ=2πfx, where *f* is the fringe frequency and *x* is the screen coordinate. However, when the phase includes information about the bottom surface, i.e., the information of *q*, its continuous phase will no longer be linear with the fringe frequency, as shown in Figure 3b. By denoting the screen coordinates of the two points as *x*_1_ and *x*_2_, respectively, then there is an offset of the point *q* related to the point *p* as Δx. When combining Equations (8) and (9), there is

(12) $$\begin{array}{cc} \frac{-\sum_{n=0}^{N-1}I_{n}\sin\delta_{n}}{\sum_{n=0}^{N-1}I_{n}\cos\delta_{n}} & =\frac{\sin\phi_{t}+\gamma \sin\phi_{b}}{\cos\phi_{t}+\gamma \cos\phi_{b}}\\ & =\frac{\left[1+\gamma \cos(2f\Delta x)]\sin(2\pi fx_{1})+\gamma \sin(2\pi f\Delta x)\cos(2\pi fx_{1}\right)}{\left[1+\gamma \cos(2f\Delta x)]\cos(2\pi fx_{1})-\gamma \sin(2\pi f\Delta x)\sin(2\pi fx_{1}\right)}\\ & =\frac{\sin(2\pi fx_{1}+\Delta \varphi )}{\cos(2\pi fx_{1}+\Delta \varphi )}\\ & =\tan(2\pi fx_{1}+\Delta \varphi ) \end{array}$$

where

(13) $$\tan\Delta \varphi =\frac{\gamma \sin(2\pi f\Delta x)}{1+\gamma \cos(2\pi f\Delta x)}$$

Therefore, the continuous phase ϕ can be expressed as

(14) $$\phi =2\pi fx_{1}+\Delta \varphi$$

and

(15) $$\Delta \varphi =\arctan\frac{\gamma \sin(2\pi f\Delta x)}{1+\gamma \cos(2\pi f\Delta x)}$$

The phase errors change along with fringe frequencies, as shown in Figure 3c. It can be seen that the phase errors are zero at certain frequencies. From Equation (15), $\Delta \varphi (f_{k})=0$ when $\;f_{k}=\frac{k}{2\Delta x}$ (k∈N), which means that the true phase of the top surface can be obtained as long as the location of the zero phase errors is detected.

With parasitic reflection, the modulation intensity is also the comprehensive result, given by

(16) $$Modul(f)=M_{1}(f)\sqrt{1+2\gamma \cos(2\pi f\Delta x)+\gamma^{2}}$$

According to Equation (16), the curve is located at the extremum when $f_{k}=\frac{k}{2\Delta x}$, which means that there are a series of *f_k_* to make the phase errors zero. But it is well known that the phase accuracy is not only related with fringe frequencies but also modulation. It is easy to see that the odd *k* is matched with the valley of the modulation curve, and the even *k* is for the peak of the curve. Therefore, the odd *k* is discarded, and the frequency *f_k_*, whose *k* satisfies maximum even, is selected to be the optimal frequency *f*_0_. When the frequencies are ranged between [1, *f_max_*], the optimal frequency $f_{0}=floor\left[f_{max}2\Delta x\right]/2\Delta x$.

#### 2.2.2. Calculating Δ*x* and *x*_1_

Principally, the minimum frequency with zero phase error can be calculated by $\frac{1}{\Delta x}$ if the phase errors are known. But the phase errors cannot be acquired directly; therefore, the derivative of Equation (12) is introduced to find the locations of zero phase errors, given by

(17) $$\phi^{\prime }(f)=2\pi x_{1}+2\pi \Delta x\frac{\gamma^{2}+\gamma \cos(2\pi f\Delta x)}{1+\gamma^{2}+2\gamma \cos(2\pi f\Delta x)}$$

**Figure 4:**
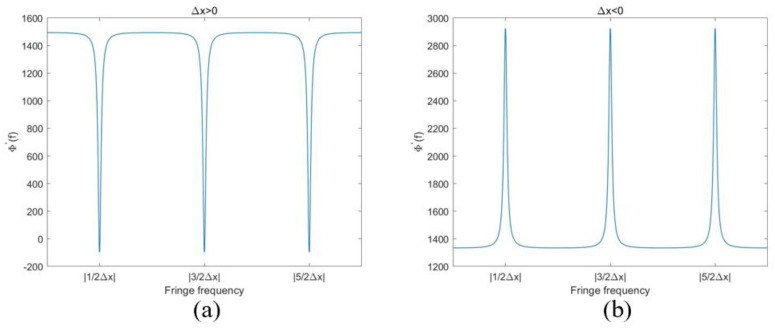
The relationship between ∆x and $\phi^{\prime }(f)$. (**a**) ∆x>0; (**b**) ∆x<0.

The even *k* is denoted as *k*_even_, the odd *k* as *k*_odd_. This function hits its extremums when $f=\frac{k_{odd}}{2∆x}$. The interval of two adjacent extremums is $\frac{1}{∆x}$, by which we can obtain *f*_0_. Figure 4 shows the ideal variation in $\phi^{\prime }(f)$ when Δ*x* > 0 or Δ*x* < 0, γ=0.9 and $f\in [0,\frac{3}{\left|∆x\right|}]$. Obviously, the valleys of ϕ′(f) are what we should detect when Δ*x* > 0; the peaks of ϕ′(f) should be detected when Δ*x* < 0. Figure 4 shows the curves with different Δ*x*. Apparently, the local extremums of ϕ′(f) can be minimum and maximum while Δ*x* is positive or negative. Then, it is not easy to decipher the sign of Δ*x* from the extremums with a uniform threshold.

**Figure 5:**
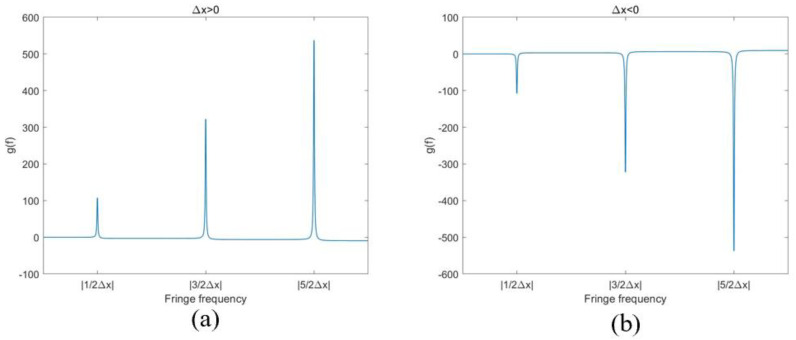
The sign relationship between ∆x and g(f): (**a**) ∆x>0 and (**b**)  ∆x<0.

Therefore, we introduced a function $g(f)=\phi (f)-f\phi^{\prime }(f)$, as shown in Figure 5. The sign of the peaks of the g(f) function is the same as the sign of Δ*x*. No matter what the symbol of Δ*x* is, the locations can always be found easily by $\left|g(f)\right|$.

Then, the optimal frequency *f*_0_ can be obtained, and the continuous phase is acquired by the temporal phase unwrapping algorithm. In practice, there is no frequency that exactly equals *f*_0_ in the fringe frequencies adopted. We need to interpolate the frequencies and the continuous phase, i.e., $f^{\prime }$ and $\phi^{\prime }$, respectively. If a frequency $f_{0}^{\prime }$ in the interpolated frequencies meet $\left|f_{0}^{\prime }-f_{0}\right|<\varepsilon$, where ε is the accuracy threshold and *ε* < 10^−5^, it can be considered the best frequency. Therefore, the screen coordinate *x*_1_ can be given by

(18) $$x_{1}=\frac{\phi_{f_{0}^{\prime }}^{\prime }}{2\pi f_{0}^{\prime }}$$

**Figure 6:**
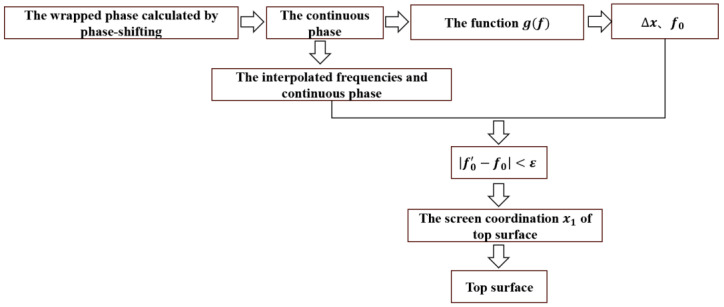
Architecture of the proposed method.

The same set of equations can be derived for the *y* direction. After obtaining the screen coordinates, the slopes of two directions can be built, and the 3D profile of the top surface can be reconstructed subsequently. The whole procedure of the proposed method for a pixel is represented in Figure 6.

## 3. Experiments

**Figure 7:**
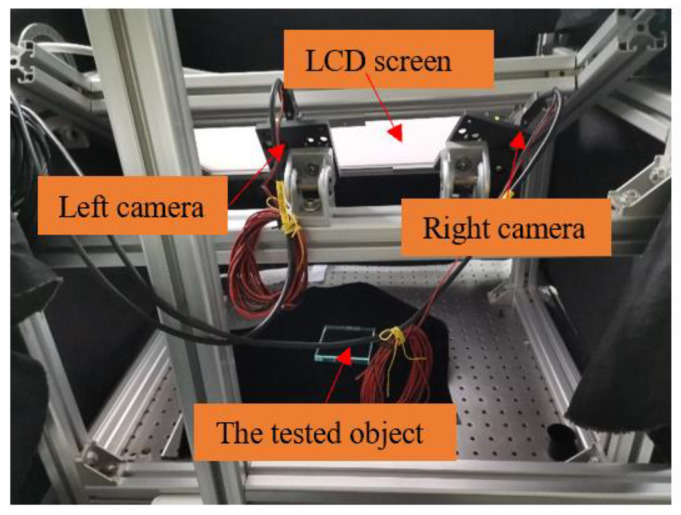
The experimental system.

The experimental system consists of a 1680 × 1050 LCD monitor with a pixel pitch of 0.258 mm and a pair of 1360 × 1024 cameras with a pixel pitch of 4.65 μm. For the monocular PMD system, the height of the tested object causes ambiguity of the surface, so two cameras are used in the experiments [22]. The cameras’ focal lengths are both 12 mm. The left camera is the main field of view. The system setup is shown in Figure 7. And the system was calibrated [23].

### 3.1. Measurement of a 40 mm Thick Standard Planar Element

Theoretically, the thicker the tested unit, the better the division between the parasitic reflections will be. Therefore, a standard planar element with a thickness of 40 mm was measured. Considering the specular characteristics of the side surfaces, they were covered to avoid unnecessary reflections. The size of the measurement region was 33 mm × 38 mm. The orthogonal fringe patterns with an inclined angle θ=45° were displayed on the screen. The frequency *f* of the fringe patterns ranged between $\left[\frac{1}{L},\frac{128}{L}\right]$, where L was the effective length of the screen. Five-step phase-shifting was used to generate the fringe patterns at 320 continuous spatial frequencies within the above frequency range. And the continuous phase was calculated by the multi-frequency hierarchical algorithm [24].

**Figure 8:**
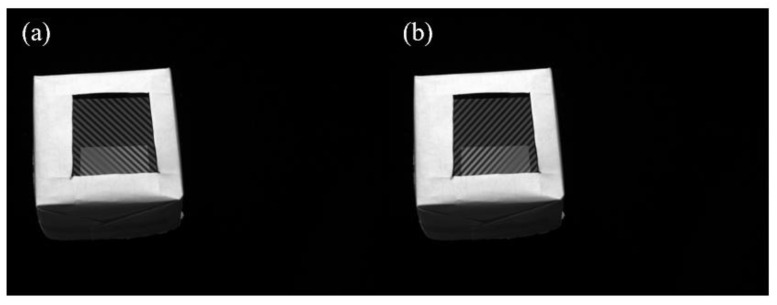
The deformed patterns captured by left camera. (**a**) The *x*−direction; (**b**) the *y*−direction.

**Figure 9:**
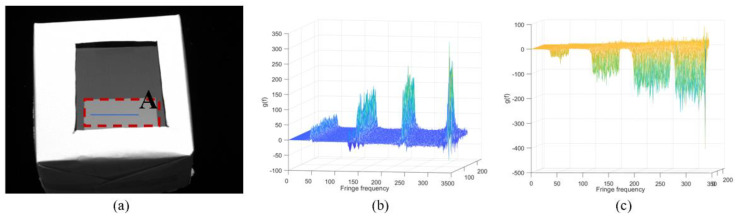
(**a**) Region A, which is the main application area of the proposed method. (**b**) The *x*−direction: the variation in function g(f) of the pixels on the line in region A. (**c**) The *y*−direction: the variation in function g(f) of the pixels on the line in region A.

**Figure 10:**
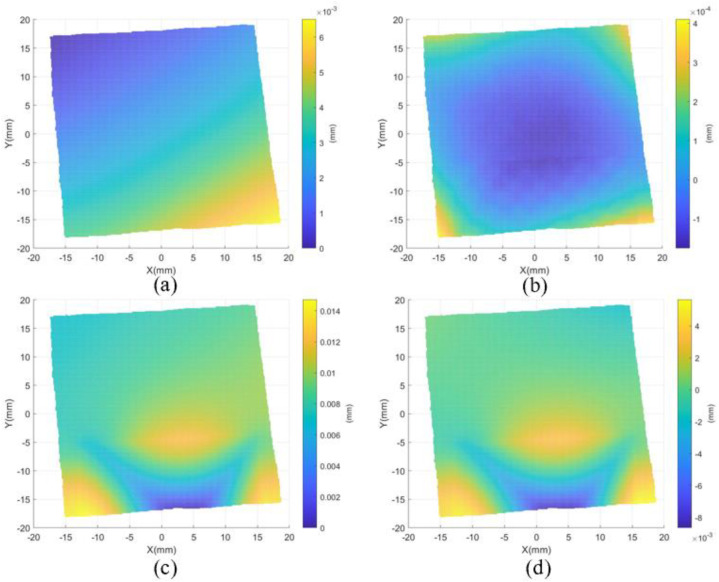
The reconstructed results. (**a**) Reconstructed top surface using the proposed method, (**b**) reconstructed result without the low−order errors using the method, (**c**) reconstructed top surface using traditional PMD, and (**d**) reconstructed result without the low−order errors using traditional PMD.

The *x*- and *y*-fringe patterns captured by the left camera at *f* = 0.0663 are shown in Figure 8. As shown in Figure 8, there are low contrast regions caused by parasitic reflection. As shown in Figure 9, region A is the main application area of the proposed method, which is framed with a red dotted line. The variation of $g\left(f\right)$ in different directions on the blue line in region A is given. There are multiple peaks of function $g\left(f\right)$ for two directions, as shown in Figure 9b,c. In order to illustrate that the proposed method successfully decouples the top surface information from the superposed patterns, the reconstructed result of the proposed method is compared with the reconstructed result of the traditional PMD method. The same unwrapping algorithm is used for the traditional PMD. The traditional PMD method reconstructed the top surface based on the phase of the highest fringe frequency. Figure 10a,c show the measurement results of the proposed method and traditional PMD, respectively. From the results, it can be clearly seen that the reconstructed error area of the traditional PMD method is mainly located in the A region, which directly illustrates the negative influence of parasitic reflection on the measurement accuracy of the traditional PMD method. The reconstructed results without low-order errors, i.e., removing a fitted plane, of the proposed method and traditional PMD are shown in Figure 10b,d, with STD=0.02 μm, PV=0.58 μm and STD=0.65 μm, PV=14.00 μm, respectively.

**Figure 11:**
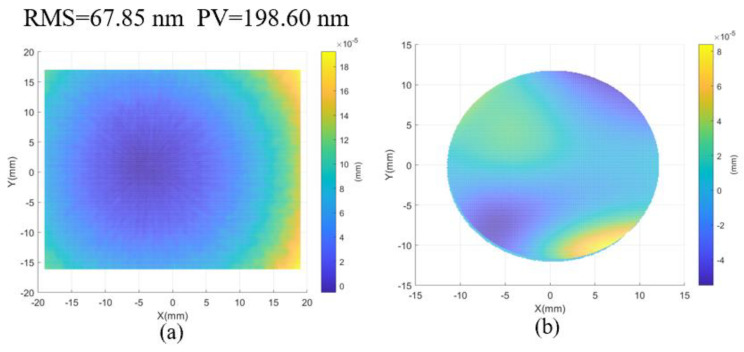
(**a**) The top surface measured by interferometer and (**b**) error map with the use of the proposed method (remove five terms of Zernike polynomials).

Further, the result measured by the interferometer is regarded as a standard value, where the PV and RMS values are 67.95 nm and 198.60 nm, respectively, as shown in Figure 11a. To further evaluate the measuring accuracy, the corresponding central circular regions are selected from the results of both the proposed method and the interferometry, which are shown in Figure 10b and Figure 11a, respectively. Then, the surface error is fitted by Zernike polynomials, and this is presented in Figure 11b. The PV value and RMSE of the error map with five terms removed are 0.14 μm and 0.02 μm.

### 3.2. Measurement of a 19 mm Flat Glass

**Figure 12:**
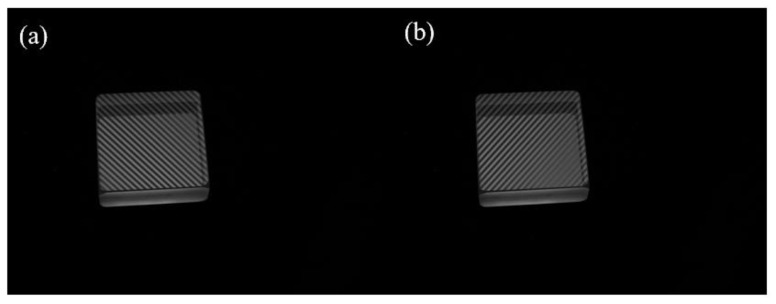
The superposed fringes captured by left camera. (**a**) x−direction; (**b**) y−direction.

**Figure 13:**
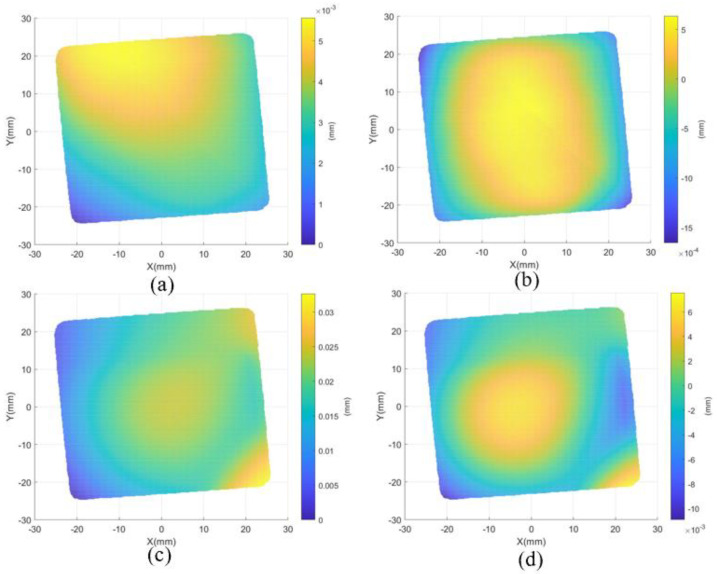
The reconstructed results of a 19 mm thick flat glass. (**a**) Reconstructed top surface using the proposed method, (**b**) reconstructed result without the low−order errors using the method, (**c**) reconstructed top surface using traditional PMD, and (**d**) reconstructed result without the low-order errors using traditional PMD.

To verify the performance of the proposed method further, a flat glass with a thickness of 19 mm was measured. The same experimental procedures were applied. The size of the measurement region was 50 mm × 50 mm. Figure 12a,b show the *x*- and *y*-fringe patterns captured by the left camera at *f* = 0.0663. The reconstructed results of the proposed method and traditional PMD are shown in Figure 13a,d. Without low-order errors, the PV and STD values of the result of the traditional PMD are 18.40 μm and 0.86 μm; the PV and STD values of the proposed method are 2.50 μm and 0.04 μm.

## 4. Discussion

### 4.1. Limitation

It can be seen from Section 2 that the performance of the proposed method is related to whether there are peaks in the curve of g(f). If there is at least one peak in this curve, the proposed method can calculate Δ*x* well; otherwise, the proposed method will fail. The frequencies are related to the number of fringes displayed on the LCD and effective length *L*. Denote the maximum number of fringes as $S_{max}$, and then minimum Δ*x* is calculated by the proposed method, which can be expressed as follows:

(19) $$\Delta x_{\min}=\frac{L}{2S_{\max}}$$

Both References [10,13] mentioned that Δ*x* is proportional to the thickness of the object. Therefore, the smaller that Δ*x* is measured at, the thinner the planar elements measured are.

### 4.2. Range of Application

**Figure 14:**
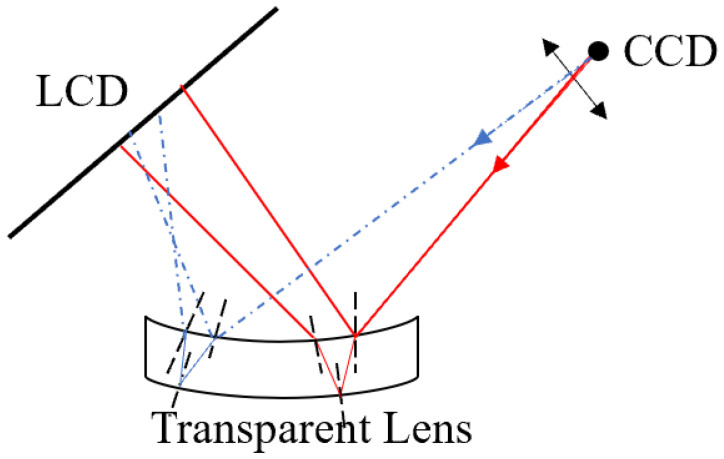
Schematic diagram of the measurement lens.

Whether it is a planar element or a lens, the intensity model is the same for a camera pixel when parasitic reflection exists, based on which the proposed method can be extended to measurement lenses theoretically. According to the law of reflection, the changes in Δ*x* for a lens are more dramatic than that for planar elements, and there may be a transition zone in these changes, as shown in Figure 14. The proposed method relies on the validity of the continuous phase. However, due to the joint influence of both the curvature of the lens and parasitic reflection, the measurement of lenses is more complicated, and the continuous phase of the lens may be problematic. The effect of curvature is not considered at this time, so the algorithm may only be suitable for transparent planar objects.

## 5. Conclusions

When measuring transparent objects, a significant error will be introduced due to inaccurate phase extraction with a traditional phase-shifting algorithm. This phenomenon is caused by parasitic reflection. In order to successfully untangle the phase of the top surface from the superposed fringes, this paper proposes a new method based on the principle of multi-frequency and phase-shifting. This method builds a mathematical model of phase errors along the fringe frequencies. By analyzing this model, it is concluded that the phase error is zero at a certain frequency at which the difference in the continuous phase between the top and the bottom surfaces is an integral multiple of 2π. To find this frequency, we introduce an auxiliary function whose location of peaks is related to this frequency. As a result, we can eliminate the effect of parasitic reflection by calculating the continuous phase at this frequency. By applying the proposed method to measure a 40 mm standard planar element, the RMSE of the reconstruction result was up to nanometers. Then a 19 mm thick flat glass was measured to verify the feasibility of the proposed method, whose reconstruction result is better than that of traditional PMD. The two experiments indicate that the proposed method can decouple the superposed patterns.

## Data Availability

Data underlying the results presented in this paper are not publicly available at this time, but they may be obtained from the authors upon reasonable request.
